# Immunizations During Pregnancy

**DOI:** 10.1155/S1064744994000104

**Published:** 1994

**Authors:** Joseph J. Apuzzio

**Affiliations:** Department of OB/GYN New Jersey Medical School Newark New Jersey USA


Infectious Diseases in Obstetrics and Gynecology 1:209 (1994)
© 1994 Wiley-Liss, Inc.

### Immunizations During Pregnancy

The time has come when physicians may be able to practice preventive medicine, which is particularly important in reference to those infectious diseases that can be prevented by immunizations. Even pregnant women should be candidates for certain immunizations. It is the scope of this editorial to cover just two of those immunizations that can be prescribed for pregnant women: the influenza vaccine and the hepatitis B vaccine.

The influenza vaccine is changed on a yearly basis to immunize against the strains expected to cause disease that season. Although the influenza vaccine is available, it is often not prescribed or used in the pregnant patient. There are several reasons for not prescribing it during pregnancy, one of which is the physician's fear of medical/legal consequences. Another reason is that patients who have a bad outcome during pregnancy may have recall bias and blame the unfortunate outcome on a specific circumstance, drug, or immunization. Although this cause and effect is almost never the case, it is certainly difficult to convince the patient of this point. However, physicians should be proactive in offering the influenza vaccine to those patients who may suffer the highest complication rates, namely, patients who are severe asthmatics or those with heart disease. Therefore, we should remind ourselves, particularly in the northern hemisphere of the United States, that in the months of October and November before the winter flu season, we should offer the influenza vaccine to patients who have entered the second trimester or more of pregnancy without fear that the vaccine will cause a fetal problem.

The hepatitis B vaccine, now offered in a recombinant form, should be made available to our female population, especially to individuals who either have a life style that may increase their risk of acquiring hepatitis B infection, such as health care workers, or live in a community where there is a high prevalence of hepatitis, such as in a large inner-city population. Unfortunately, the cost of the hepatitis B vaccine for an adult is approximately $50 per dose, with each patient requiring 3 doses over a course of 6 months. For this reason, the cost factor has limited the use of the hepatitis B vaccine. With the current active support of the Academy of Pediatrics to immunize newborns and children with the hepatitis B vaccine, many individuals may be immunized in the future. However, until that point is reached, we should offer immunization to women who are considered high risk, preferably before they get pregnant, and remember that pregnancy is not a contraindication for the hepatitis B vaccine.

*Joseph J. Apuzzio, M.D.*
*Department of OB/GYN*
*New Jersey Medical School*
*Newark, New Jersey*

